# Baseline Disease Activity and Moderate-to-High Disease Activity at 6 Months in Rheumatoid Arthritis-Associated Interstitial Lung Disease: A Multicentre Real-World Study from the ANSWER Cohort

**DOI:** 10.3390/jcm15218267

**Published:** 2026-10-23

**Authors:** Ayaka Yoshikawa, Takuya Kotani, Wataru Yamamoto, Kenichiro Hata, Koji Nagai, Hideyuki Shiba, Yuri Hiramatsu, Yumiko Wada, Koichi Murata, Hideo Onizawa, Kohei Tsujimoto, Iku Shirasugi, Masanori Tsubosaka, Motomu Hashimoto, Masao Katsushima, Yonsu Son, Naofumi Yoshida, Yuji Nozaki, Chisato Ashida, Tohru Takeuchi

**Affiliations:** 1 Division of Rheumatology, Department of Internal Medicine (IV), Osaka Medical and Pharmaceutical University, Takatsuki 569-8686, Osaka, Japan; ayaka.yoshikawa@ompu.ac.jp (A.Y.); kenichirou.hata@ompu.ac.jp (K.H.); kouji.nagai@ompu.ac.jp (K.N.); hideyuki.shiba@ompu.ac.jp (H.S.); yuri.hiramatsu@ompu.ac.jp (Y.H.); yumiko.wada@ompu.ac.jp (Y.W.); tooru.takeuchi@ompu.ac.jp (T.T.); 2 Department of Health Information Management, Kurashiki Sweet Hospital, Kurashiki 710-0051, Okayama, Japan; wataru.yamamoto@answer-cc.jp; 3 Department of Advanced Medicine for Rheumatic Diseases, Graduate School of Medicine, Kyoto University, Kyoto 606-8501, Kyoto, Japan; kchm@kuhp.kyoto-u.ac.jp (K.M.); honizawa0102@gmail.com (H.O.); 4 Department of Respiratory Medicine and Clinical Immunology, Graduate School of Medicine, The University of Osaka, Suita 565-0871, Osaka, Japan; k.tsujimoto@imed3.med.osaka-u.ac.jp; 5 Department of Rheumatology and Clinical Immunology, Kobe University Graduate School of Medicine, Kobe 650-0017, Hyogo, Japan; shira19@med.kobe-u.ac.jp; 6 Department of Orthopaedic Surgery, Kobe University Graduate School of Medicine, Kobe 650-0017, Hyogo, Japan; masanor1@med.kobe-u.ac.jp; 7 Department of Clinical Immunology, Graduate School of Medicine, Osaka Metropolitan University, Osaka 545-8585, Osaka, Japan; motomuhashimoto@gmail.com (M.H.); m22647i@omu.ac.jp (M.K.); 8 First Department of Internal Medicine, Kansai Medical University, Hirakata 573-1010, Osaka, Japan; y-son@n-watanabe-hosp.jp (Y.S.); naochi9@gmail.com (N.Y.); 9 Department of Hematology and Rheumatology, Kindai University School of Medicine, Osakasayama 589-8511, Osaka, Japan; yuji0516@med.kindai.ac.jp (Y.N.); c-ashida@med.kindai.ac.jp (C.A.)

**Keywords:** rheumatoid arthritis, interstitial lung disease, disease activity, DAS28-ESR, age, real-world data, ANSWER cohort

## Abstract

**Background/Objectives**: In rheumatoid arthritis-associated interstitial lung disease (RA-ILD), factors associated with moderate-to-high disease activity (MDA/HDA) during follow-up are not well defined. We examined baseline factors associated with MDA/HDA at 6 months. **Methods**: We analyzed 278 patients with RA-ILD from the multicentre ANSWER cohort. At 6 months, patients were classified by the 28-joint Disease Activity Score using erythrocyte sedimentation rate (DAS28-ESR) as remission/low disease activity (LDA) or MDA/HDA. Logistic regression used age and baseline DAS28-ESR as continuous variables (*n* = 178 with complete baseline DAS28-ESR); discrimination, calibration and optimism (1000 bootstrap resamples) were assessed. A sensitivity analysis used the ESR-free Clinical Disease Activity Index (CDAI) as the outcome. **Results**: In all, 148 patients achieved remission/LDA and 130 had MDA/HDA. Baseline DAS28-ESR was independently associated with MDA/HDA (odds ratio 2.01 per point, 95% CI 1.57–2.57), as was age (1.05 per year, 1.01–1.08). The area under the curve was 0.768 for DAS28-ESR alone and 0.776 with age added; internal validation of the two-variable model showed little estimated optimism (corrected area 0.772, calibration slope 0.984, 95% CI 0.693–1.396). With the CDAI-based outcome, the association with age was not reproduced (odds ratio 1.00, 0.97–1.04). **Conclusions**: Baseline disease activity is strongly associated with MDA/HDA at 6 months in RA-ILD. The incremental value of age was small, and the association with age was not reproduced when the outcome was defined by the CDAI. The age finding should therefore be regarded as secondary and dependent on the disease activity measure used. External and prospective validation is required before these findings are applied clinically.

## 1. Introduction

Rheumatoid arthritis (RA) is a systemic autoimmune disease characterized by synovial inflammation and progressive joint destruction, affecting approximately 1% of the global population [1]. Beyond joint manifestations, RA commonly involves extra-articular organs, with pulmonary complications among the most serious. Interstitial lung disease (ILD) develops in about 10–30% of patients with RA, and it is a major contributor to poor survival outcomes [2], with reported 5-year survival rates of approximately 60% [3,4,5,6,7].

The pharmacological management of RA-related ILD (RA-ILD) is complicated by uncertainty about the pulmonary safety of anchor drugs. A distinction should be drawn between acute drug-induced pulmonary toxicity, such as methotrexate (MTX) pneumonitis, which is a well-recognized although uncommon event, and long-term progression of pre-existing RA-ILD, for which the evidence is far less consistent. Earlier reports suggested that MTX and tumor necrosis factor (TNF) inhibitors might worsen pulmonary lesions [8], and this concern has shaped prescribing practice for many years. More recent observational data, however, have not confirmed an increased risk of ILD progression with MTX, and some analyses have reported a neutral or even favourable association [9,10]. The safety of TNF inhibitors in established RA-ILD remains similarly unsettled [11]. As a result, physicians treating RA-ILD often face a therapeutic trade-off that is based on incomplete evidence, and treatment intensity may be limited in ways that are not always supported by current data. Known prognostic factors for poor RA-ILD survival include advanced age, male sex, smoking history, and the presence of a usual interstitial pneumonia pattern [12].

Emerging evidence suggests high RA disease activity may worsen ILD outcomes [13,14]. Suppression of joint disease activity has therefore been proposed as a treatment goal that may also be relevant to pulmonary outcomes [7], although this relationship has been described in observational studies and should be regarded as an association rather than a demonstrated causal effect. However, it remains unclear which baseline clinical characteristics identify patients with RA-ILD who are less likely to be in remission or low disease activity (LDA) during follow-up.

Age and baseline disease activity are established prognostic factors in RA in general. What is not known is whether they behave in the same way in RA-ILD, a population in which treatment intensity is frequently constrained by pulmonary considerations and in which the achievable rate of remission may therefore differ. The present study addresses this specific question. Using comprehensive real-world data from the multicentre ANSWER cohort, we examined which baseline factors are associated with moderate-to-high disease activity (MDA/HDA) at 6 months in patients with RA-ILD and examined their discriminative performance and internal validity. Our aim was to characterize these associations, not to derive treatment recommendations.

## 2. Materials and Methods

### 2.1. Study Design and Patients

This retrospective cohort study used data from the Kansai Consortium for Well-Being of Rheumatic Disease Patients (ANSWER), a multicentre observational registry of patients with RA in the Kansai region of Japan. Data of patients treated between January 2009 and October 2023 were analyzed. Nine institutions participated in the ANSWER cohort: Kyoto University, Osaka University, Osaka Medical and Pharmaceutical University, Kansai Medical University, Kobe University, Nara Medical University, Osaka Metropolitan University, Kindai University, and Osaka Red Cross Hospital. Information allowing the presence or absence of ILD to be ascertained was available at six of these institutions, and the present analysis was therefore restricted to patients registered at those six centres.

The study was conducted in accordance with the Declaration of Helsinki. The ANSWER registry has been approved by the institutional review board of each participating institution: Kyoto University (approval no. R053, 24 March 2016), Osaka Medical and Pharmaceutical University (approval no. 1526, 14 July 2014), Osaka University (approval no. 15300, 4 November 2015), Osaka Metropolitan University (approval no. 2021-074, 9 June 2021), Kansai Medical University (approval no. 2014625, 21 November 2017), Kobe University (approval no. 1738, 20 March 2015), Nara Medical University (approval no. 1692, 23 January 2018), Kindai University (approval no. R05-129, 24 October 2023), and Osaka Red Cross Hospital (approval no. 644, 1 September 2015). The present retrospective analysis was approved by the Institutional Review Board of Osaka Medical and Pharmaceutical University on 10 November 2023 (approval no. 2023-145).

Eligible patients met either the 1987 American College of Rheumatology (ACR) classification criteria [15] or the 2010 ACR/European League Against Rheumatism (EULAR) classification criteria for RA [16]. Antirheumatic therapies, including biological DMARDs (bDMARDs) and Janus kinase inhibitors (JAKi), were prescribed according to the Japan College of Rheumatology guidelines [17].

Baseline was defined as the first visit at which patients were registered in the ANSWER cohort. Registration did not necessarily coincide with the initiation of a new antirheumatic therapy; patients already receiving stable treatment could also be registered. Baseline medication data therefore represent prevalent rather than newly initiated therapy, and this study should not be interpreted as an analysis of response to a defined treatment start. The following demographic and clinical variables were collected at baseline: age, sex, RA disease duration, smoking history, rheumatoid factor (RF) positivity and titre, anti-cyclic citrullinated peptide antibody (ACPA) positivity and titre, and disease activity indices, including Disease Activity Score in 28 joints using C-reactive protein (DAS28-CRP), DAS28 using erythrocyte sedimentation rate (DAS28-ESR), Simplified Disease Activity Index (SDAI), and Clinical Disease Activity Index (CDAI). Serum levels of Krebs von den Lungen-6 (KL-6) and matrix metalloproteinase-3 (MMP-3) were obtained. Treatment-related data included doses of MTX, glucocorticoids (prednisolone-equivalent), other conventional synthetic DMARDs (csDMARDs; including sulfasalazine, bucillamine, iguratimod, and tacrolimus), bDMARDs (such as TNF inhibitors, IL-6 receptor inhibitors, and CTLA4-Ig), and JAKi.

ILD was diagnosed using chest radiography and/or high-resolution computed tomography (HRCT) findings interpreted by rheumatologists, orthopaedic surgeons, and/or radiologists at each centre, according to routine clinical practice. As this is a real-world multicentre registry, no standardized radiological criteria were prescribed across centres and no central adjudication of images was performed. HRCT pattern, extent of fibrosis, forced vital capacity, and diffusing capacity were not collected in the registry and were therefore unavailable for analysis. These constraints are addressed in the Limitations. Patients with incomplete 6-month disease activity data were excluded.

### 2.2. Outcome

The outcome was disease activity status at 6 months after baseline, assessed using the DAS28-ESR. According to the EULAR criteria, patients were categorized into the remission, LDA, moderate disease activity (MDA), or high disease activity (HDA) groups: remission (DAS28-ESR < 2.6), LDA (2.6 ≤ DAS28-ESR < 3.2), MDA (3.2 ≤ DAS28-ESR ≤ 5.1), and HDA (DAS28-ESR > 5.1) [18]. For analysis, patients were dichotomized into those achieving remission/LDA and those with MDA/HDA at 6 months. Because baseline disease activity was not a condition for entry, MDA/HDA at 6 months denotes the disease activity state at that time point and is not described as persistent disease activity or as treatment failure. Both assessment dates were recorded for 207 patients, in whom the median interval between the baseline and 6-month assessments was 182 days (interquartile range 170–196); 98.6% of intervals fell between 90 and 270 days.

### 2.3. Candidate Variables

Candidate baseline variables were age, sex, RA disease duration, smoking history, RF and ACPA status and titres, HAQ, KL-6 and MMP-3 levels, and disease activity indices. These were selected because they are routinely available in clinical practice and have been reported as prognostic factors in RA. Treatment variables were recorded and compared between outcome groups but were not entered into the prediction models because baseline medication reflects prevalent therapy and treatment changes during follow-up were not captured by the registry; including them would confound rather than clarify the association between baseline status and 6-month outcome.

### 2.4. Statistical Analysis

Continuous data are reported as medians with interquartile ranges, whereas categorical data are presented as counts and percentages. Group comparisons were performed using the Mann–Whitney *U* test for continuous measures and Fisher’s exact test for categorical measures. Statistical significance was defined as a two-sided *p*-value < 0.05.

Associations between baseline variables and MDA/HDA at 6 months were first examined in univariable logistic regression models. The primary multivariable model included age and baseline DAS28-ESR, both retained as continuous variables. These variables were selected on clinical grounds: age is an established prognostic factor in RA in general, and baseline disease activity was the principal exposure of interest in this study; both are also available at every routine consultation. Other disease activity indices were not entered simultaneously because they share components with DAS28-ESR and are strongly correlated with it. Analyses were conducted on complete cases, and the number of patients contributing to each model is reported.

Discrimination was quantified by the area under the receiver operating characteristic curve (AUC), with 95% confidence intervals (CI) obtained from 2000 bootstrap resamples. Calibration was assessed by plotting observed against predicted probabilities and by the calibration slope. Three models were compared: age alone, baseline DAS28-ESR alone, and the two variables together as continuous predictors; the incremental contribution of age was tested by a likelihood-ratio test. Optimism arising from developing and evaluating the model in the same data was estimated by 1000 bootstrap resamples, from which an optimism-corrected AUC and calibration slope were derived.

Separately from these models, a simple 0/1/2 count was examined, in which patients were assigned one point for baseline DAS28-ESR ≥ 3.8 and one point for age ≥ 58 years. The cut-off values were determined as the values maximizing the Youden index on the ROC curve and are therefore data-derived rather than prespecified. This count is reported as an exploratory descriptive analysis only, in Section 3.6; it was not entered into the model comparison above and is not proposed as a clinical stratification tool.

Because ESR rises with age independently of inflammatory activity, an association between age and a DAS28-ESR-based outcome could partly reflect the measurement properties of the index rather than poorer disease control. To examine this, we repeated the analysis using the CDAI, which contains neither ESR nor C-reactive protein, defining the 6-month outcome as CDAI > 10 versus CDAI ≤10. Because this outcome does not require a 6-month DAS28-ESR value, the analysis was not confined to the 278 patients who formed the primary analysis population: it was carried out in every patient with RA-ILD who had a baseline CDAI, a 6-month CDAI and a recorded age. This gave 240 patients: more than the 178 who formed the regression sample of the primary analysis, but fewer than the 278 of the primary analysis population, and only partly overlapping with either. Because this analysis differs from the primary analysis both in the disease activity index used to define the outcome and in the patients included, a difference in the association with age between the two cannot be attributed specifically to the erythrocyte sedimentation rate.

All statistical analyses were performed using JMP Pro version 17.0 (SAS Institute Inc., Cary, NC, USA), with model comparison, bootstrap validation, and calibration performed in Python 3.14 (NumPy 2.x).

### 2.5. Use of Generative Artificial Intelligence

**Figure 1:**
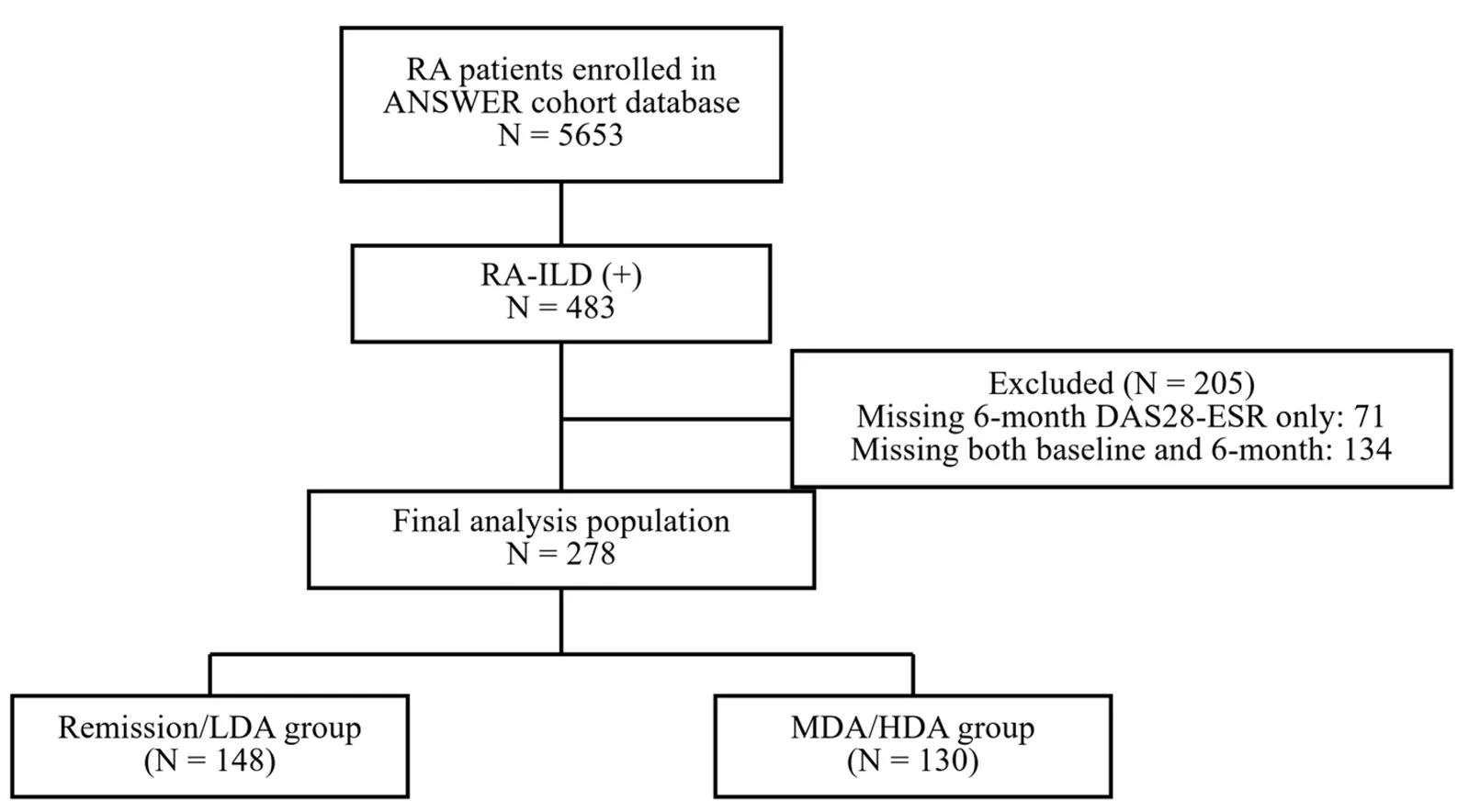
Flow diagram of patient selection. Of 5653 patients with rheumatoid arthritis (RA) enrolled in the ANSWER cohort, 483 were identified as having RA-associated interstitial lung disease (RA-ILD). After excluding 205 patients without a 6-month DAS28-ESR value, 278 patients formed the final analysis population. DAS28-ESR, Disease Activity Score in 28 joints using erythrocyte sedimentation rate; LDA, low disease activity; MDA/HDA, moderate/high disease activity.

**Figure 2:**
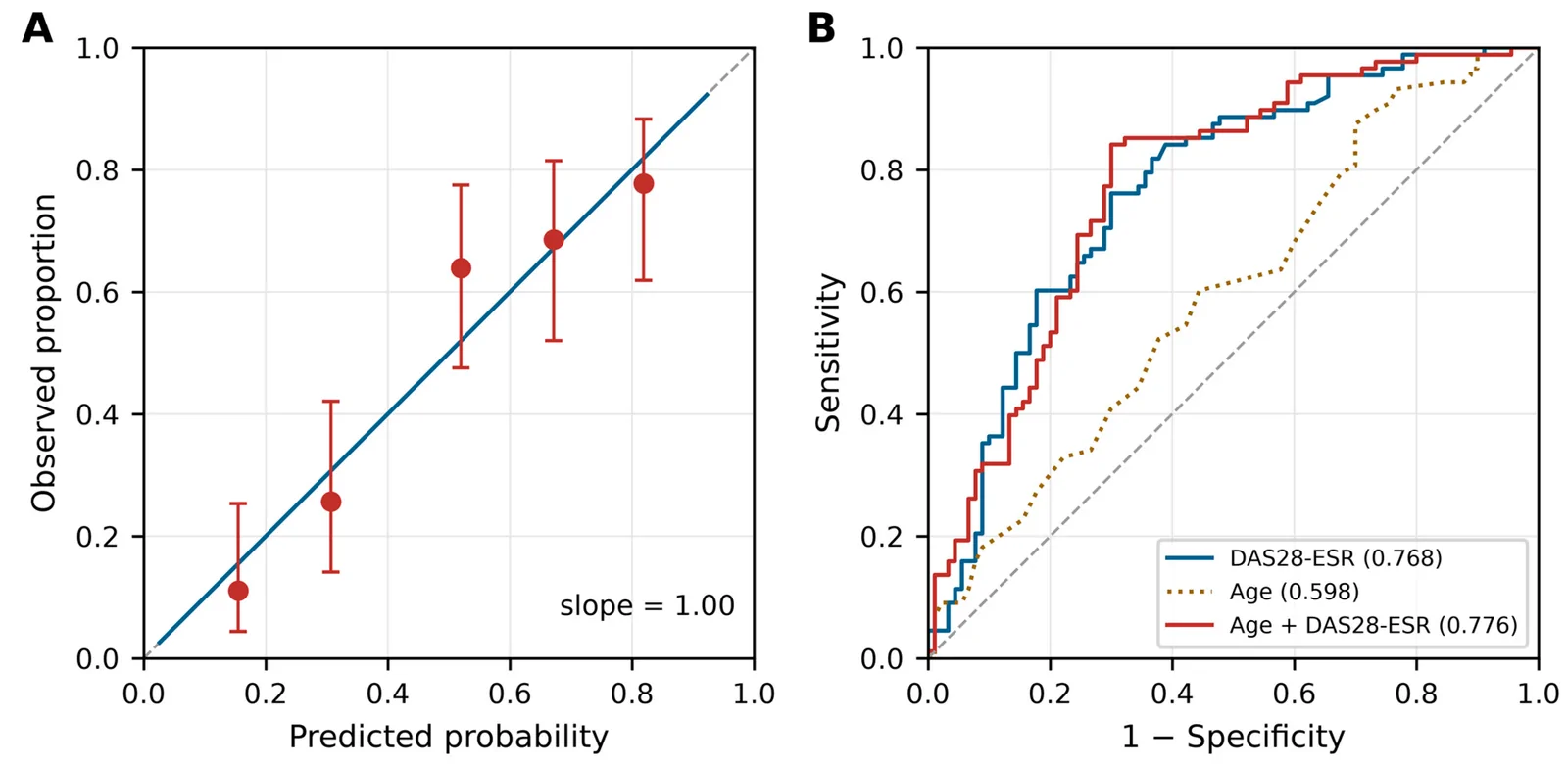
Calibration and discrimination of the models (*n* = 178). (**A**) Calibration plot for the two-variable continuous model (age and baseline DAS28-ESR). Points show observed proportions by quintile of predicted probability with 95% confidence intervals; the solid line is the fitted calibration curve and the dashed line indicates perfect calibration. (**B**) Receiver operating characteristic curves for the three models, with the area under the curve given in parentheses. DAS28-ESR, Disease Activity Score in 28 joints using erythrocyte sedimentation rate.

**Figure 3:**
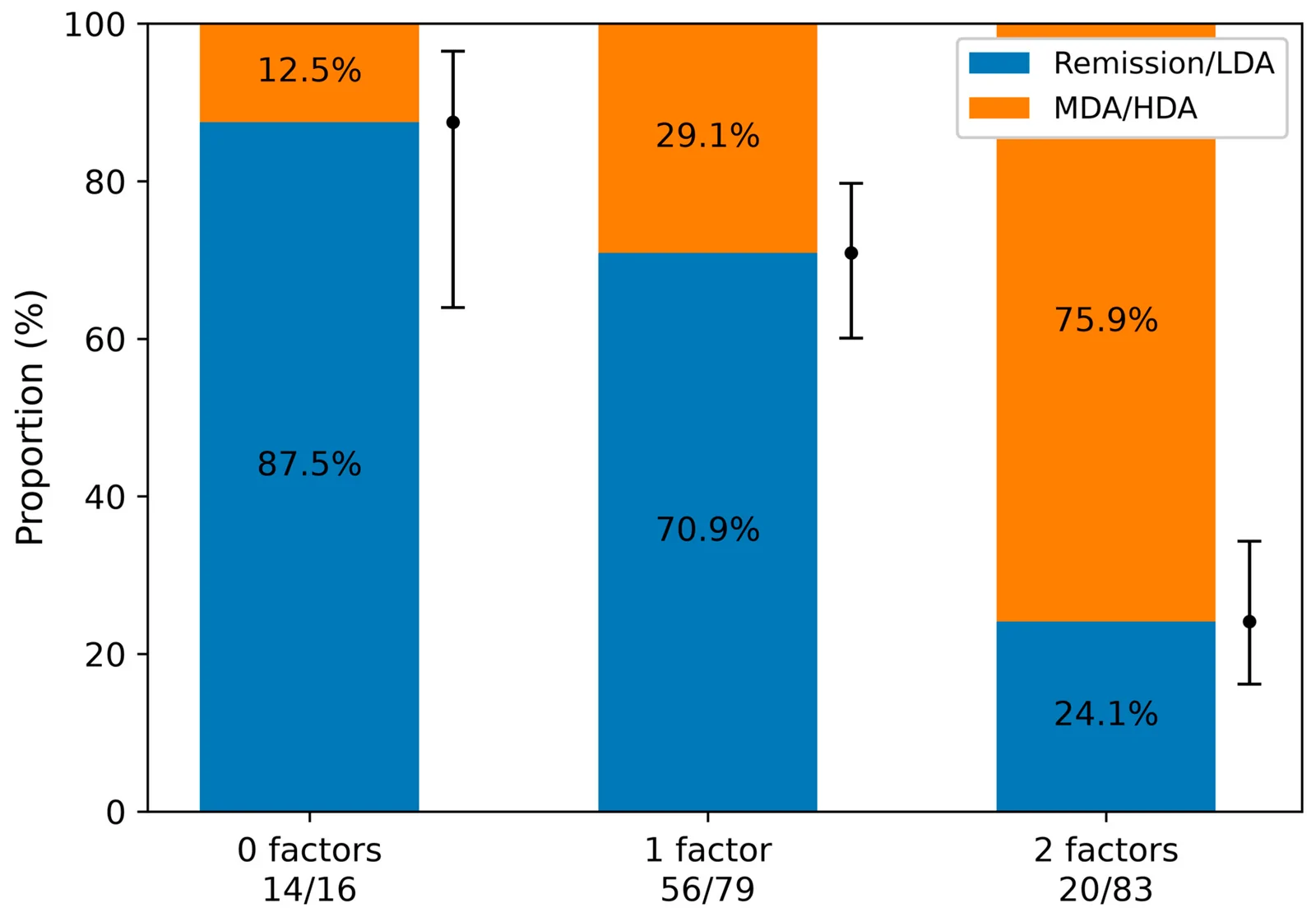
Exploratory descriptive analysis. Proportion achieving remission or low disease activity (LDA) at 6 months according to the number of baseline factors present (baseline DAS28-ESR ≥ 3.8 and age ≥ 58 years), among the 178 patients with a complete baseline DAS28-ESR. Both cut-off values were derived from the present data, and this analysis is not proposed as a clinical stratification tool. Numerators and denominators are shown below each category, and error bars indicate 95% confidence intervals for the remission/LDA proportion. DAS28-ESR, Disease Activity Score in 28 joints using erythrocyte sedimentation rate; MDA/HDA, moderate/high disease activity.

Generative artificial intelligence Claude Opus 4.1 (Anthropic) was used to assist with code generation and implementation for the additional statistical analyses reported in Section 3.4 and Section 3.5, with the preparation of Figure 1, Figure 2 and Figure 3 from the authors’ own data, with conversion of the manuscript into the journal template, and with language editing. All analytic procedures, outputs, and interpretations were independently reviewed and verified by the authors. No study data, results, or scientific interpretation were generated by artificial intelligence, and the authors take full responsibility for the content of this manuscript.

## 3. Results

### 3.1. Patient Selection and Baseline Characteristics

Of the 5653 patients with RA enrolled in the ANSWER cohort, 483 were identified as having RA-ILD. Of these, 205 were excluded because DAS28-ESR at 6 months was unavailable (6-month value missing only, 71 patients; both baseline and 6-month values missing, 134 patients), leaving 278 patients in the final analysis population (Figure 1). Age, sex, and RA disease duration did not differ appreciably between the 278 included and the 205 excluded patients (Table S1), arguing against substantial selection on these characteristics.

Baseline characteristics and initial treatments of these 278 patients are summarized in Table 1. The median age was 71 years, and 68.0% were female. The median disease duration was 14.4 years, and 43.1% had a history of smoking. RF and ACPA were positive in 84.6% and 85.1% of patients, respectively, with median titres of 91 IU/mL and 130 U/mL. The median DAS28-ESR was 3.94, MMP-3 was 110.7 ng/mL, and KL-6 was 411 U/mL. MTX and glucocorticoids (prednisolone-equivalent) were used in 23.4% and 35.3% of patients, respectively. TNF inhibitors, interleukin-6 (IL-6) receptor inhibitors, CTLA4-Ig, and JAKi were each used in 6–11% of patients.

Data were not complete for all variables. Baseline DAS28-ESR was available in 178 of the 278 patients (64%); the corresponding figures were 197 (71%) for CDAI, 184 (66%) for MMP-3, 153 (55%) for HAQ, and 111 (40%) for KL-6. Age, sex, and the 6-month outcome were available for all 278 patients. The number of patients contributing to each analysis is stated throughout.

### 3.2. Comparison Between the Remission/LDA and MDA/HDA Groups

At 6 months, 148 patients achieved remission/LDA, whereas 130 had MDA/HDA. Table 2 summarizes the baseline characteristics and treatment profiles of both groups. Compared with the remission/LDA group, the MDA/HDA group was older (median 72 vs. 69 years, *p* = 0.0182) and included more women (75.4% vs. 61.5%, *p* = 0.0146). Functional disability, measured using the Health Assessment Questionnaire (HAQ), was more pronounced in the MDA/HDA group (median 1.06 vs. 0.5, *p* < 0.001). The MDA/HDA group also had higher RF positivity (90.9% vs. 79.0%, *p* = 0.0095) and titres (median 108.8 vs. 67.4 IU/mL, *p* = 0.0057) and higher MMP-3 levels (155.7 vs. 91.3 ng/mL, *p* = 0.0019), whereas ACPA positivity and titres did not differ significantly. Smoking history did not differ between the groups (48/122, 39.3% vs. 61/131, 46.6%; *p* = 0.2558; 253 patients with available data).

Baseline disease activity scores were consistently higher in the MDA/HDA group: DAS28-CRP (3.86 vs. 2.05, *p* < 0.001), DAS28-ESR (4.79 vs. 3.06, *p* < 0.001), SDAI (17.6 vs. 6.8, *p* < 0.001), and CDAI (14.6 vs. 6.0, *p* < 0.001). No significant differences were observed between the two groups in the use of MTX, prednisolone, or biologic/targeted synthetic DMARDs.

### 3.3. Baseline Factors Associated with MDA/HDA at 6 Months

Univariable logistic regression identified several baseline factors associated with MDA/HDA at 6 months (Table 3). Higher baseline DAS28-ESR was associated with the outcome (odds ratio [OR] 1.989 per point; 95% CI 1.561–2.534; *p* < 0.0001; *n* = 178), as were higher HAQ (OR 2.537; 1.628–3.954; *p* < 0.0001; *n* = 153) and higher CDAI (OR 1.087 per point; 1.050–1.125; *p* < 0.0001; *n* = 197). Odds ratios per unit are not comparable across variables measured on different scales, and no ranking of the strength of association is implied. Greater age (OR 1.040 per year; 1.013–1.067; *p* = 0.0029; *n* = 278), female sex (OR 1.918; 1.142–3.222; *p* = 0.0138; *n* = 278), longer disease duration (OR 1.026 per year; 1.004–1.049; *p* = 0.022; *n* = 256) and higher RF (OR 1.015 per 10 IU/mL; 1.005–1.025; *p* = 0.0047; *n* = 259) were also associated with the outcome. Smoking history was not associated with the outcome (OR 0.744; 0.452–1.227; *p* = 0.247; *n* = 253).

In the multivariable model containing age and baseline DAS28-ESR as continuous variables (*n* = 178; 88 events; 44 events per variable), both remained independently associated with MDA/HDA at 6 months: baseline DAS28-ESR OR 2.006 per point (95% CI 1.568–2.567; *p* < 0.0001) and age OR 1.046 per year (1.010–1.084; *p* = 0.0125). The fitted model was logit(*p*) = −5.894 + 0.045 × age + 0.696 × DAS28-ESR.

### 3.4. Discrimination, Calibration, and Internal Validation

Discrimination and calibration of the three models are shown in Table 4 and Figure 2. Baseline DAS28-ESR alone achieved an AUC of 0.768 (95% CI 0.699–0.835). Adding age increased the AUC to 0.776 (0.700–0.846), an absolute increment of 0.008; the likelihood-ratio test for the addition of age was statistically significant (χ^2^ = 6.78, 1 df, *p* = 0.0092), but the gain in discrimination was small. Age alone discriminated poorly (AUC 0.598; 0.514–0.679).

Observed and predicted probabilities for the two-variable continuous model lay close to the line of identity across the range of predicted values (Figure 2A), with an apparent calibration slope of 1.000. Internal validation using 1000 bootstrap resamples gave an apparent AUC of 0.7761, an estimated optimism of 0.0046 and an optimism-corrected AUC of 0.7715; these three figures are reported to four decimal places so that the subtraction is apparent. The optimism-corrected calibration slope was 0.984 (95% CI 0.693–1.396): the point estimate is close to 1, but its confidence interval is wide, so the precision with which calibration could be assessed in this dataset is limited. Bootstrap internal validation therefore suggested limited optimism in this dataset. It does not establish that the model is free of overfitting, and it cannot substitute for external validation.

### 3.5. Sensitivity Analysis Using CDAI

To examine whether the association with age reflected the age dependence of ESR, the analysis was repeated using CDAI, which includes neither ESR nor C-reactive protein, to define the 6-month outcome (CDAI > 10 versus ≤ 10). Because this outcome does not require a 6-month DAS28-ESR value, the analysis was not restricted to the 278 patients of the primary analysis population: it included all 240 patients with RA-ILD who had a baseline CDAI, a 6-month CDAI and a recorded age, of whom 55 had a 6-month CDAI above 10. Of these 240 patients, 196 also belonged to the primary analysis population and 44 did not, having no 6-month DAS28-ESR value. Of the 197 patients in the primary analysis population who had a baseline CDAI, 196 therefore entered this analysis; the one remaining patient had no 6-month CDAI. Baseline CDAI remained strongly associated with the outcome (OR 1.096 per point; 95% CI 1.061–1.131; *p* < 0.0001), and the model discriminated well (AUC 0.788; 0.722–0.847). In contrast, the association with age was not reproduced (OR 1.001 per year; 0.969–1.035; *p* = 0.934); age alone did not discriminate better than chance (AUC 0.514). The association with age was therefore not reproduced when the outcome was defined without ESR. The age dependence of ESR is one possible explanation, but this analysis differs from the primary analysis both in the index used to define the outcome and in the patients included, so the difference cannot be attributed specifically to the ESR, and the mechanism cannot be established from these data.

### 3.6. Exploratory Descriptive Analysis by Baseline DAS28-ESR and Age

The following analysis is exploratory and descriptive. Because both cut-off values were derived from the present data and the incremental contribution of age was not reproduced with an ESR-free outcome, the results below are not put forward as a clinical stratification tool. Patients were assigned one point for baseline DAS28-ESR ≥ 3.8 and one point for age ≥ 58 years, the values that maximized the Youden index. The count could be applied to the 178 patients with a complete baseline DAS28-ESR. The proportion achieving remission/LDA at 6 months was 87.5% (14/16; 95% CI 64.0–96.5) among patients with no factor, 70.9% (56/79; 60.1–79.7) with one factor, and 24.1% (20/83; 16.2–34.3) with both (Figure 3). The confidence interval for the zero-factor group is wide because only 16 patients had neither factor, which is expected in a cohort with a median age of 71 years; this estimate should therefore be interpreted with caution. For completeness, the count itself discriminated with an area under the curve of 0.760 (95% CI 0.696–0.825), below that of baseline DAS28-ESR used as a continuous variable (0.768), which is consistent with the information lost through dichotomization and equal weighting. It was not included in the model comparison in Section 3.4 and is not proposed for clinical use.

## 4. Discussion

In this multicentre real-world cohort of patients with RA-ILD, baseline disease activity was strongly associated with moderate-to-high disease activity at 6 months. Each one-point increase in baseline DAS28-ESR approximately doubled the odds of MDA/HDA, and baseline DAS28-ESR alone discriminated with an AUC of 0.768. Internal bootstrap validation suggested limited optimism, although it cannot by itself exclude overfitting.

Our findings on age deserve careful interpretation. Age was statistically associated with the outcome in the DAS28-ESR-based analysis, consistent with previous reports in RA in general [19,20], in which older patients achieve remission less often because of immunosenescence, comorbidities, and constrained therapeutic options [21,22]. However, its incremental predictive value was limited: adding age to baseline DAS28-ESR increased the AUC by only 0.008. More importantly, when the outcome was defined by CDAI, which contains neither ESR nor C-reactive protein, the association with age disappeared entirely, although that analysis was conducted in a different population that was larger than the primary regression sample but smaller than the overall primary analysis population. ESR increases with age independently of inflammatory activity, and this measurement property of DAS28-ESR is a plausible explanation for at least part of the apparent age effect; our data are consistent with it but cannot establish it, and we therefore do not claim that the age effect has been accounted for. We therefore do not present age as an independent clinical risk factor in RA-ILD, and the threshold of 58 years should be regarded as a data-derived value that shifted to 61 years in the subset with complete baseline data rather than as a clinically established definition of older age.

The exploratory 0/1/2 count described patients with markedly different 6-month outcomes, from 87.5% to 24.1% achieving remission/LDA, but we do not propose it for clinical use. Its AUC of 0.760 was lower than that of baseline DAS28-ESR used as a continuous variable, reflecting the information lost through dichotomization and through the equal weighting of two predictors whose regression coefficients differ substantially. Both cut-off values were derived from the present data, and the age threshold was unstable, shifting from 58 to 61 years between analysis sets. Given also that the contribution of age was not reproduced with an ESR-free outcome, an equally weighted combination of age and disease activity cannot be justified as a clinical tool. We therefore report this analysis as exploratory and descriptive only.

Our findings are consistent with a substantial body of evidence in RA generally. Disease activity early in the course of treatment has repeatedly been shown to predict later radiographic joint damage and functional status [23,24,25,26,27], and the level of response at 3 months predicts attainment of treatment targets at 6 months [28]; very early RA has similarly been identified as a predictor of remission [29]. Our results extend this principle to RA-ILD, a population in which treatment intensity is frequently constrained, and indicate that the same baseline signal carries information there. This reasoning underlies the treat-to-target framework recommended for RA in general [30], although our data do not test whether such a strategy alters outcomes in RA-ILD.

The presence of ILD constrains treatment intensity in RA and is associated with sustained disease activity [31,32]. Contributing factors reported in the literature include reduced use of MTX and TNF inhibitors [11,33,34,35], cautious immunosuppressant administration to minimize infection risk [8,36,37], and conservative approaches shaped by multidisciplinary evaluation [8,34,35]. In our cohort, MTX was used in 23.4% of patients, well below what would be expected for an anchor drug in RA, although those who did receive it were given a median dose of 8 mg/week, which is within the range commonly used in Japanese clinical practice. We cannot determine from these data why MTX use was low. One plausible explanation is that prescribing was influenced by long-standing concerns about pulmonary toxicity, concerns that more recent evidence has called into question [9,10]. Because ILD severity, HRCT pattern, and pulmonary function were not available, we cannot establish whether MTX was preferentially withheld from patients with more extensive disease, and we make no such claim.

Sustained high disease activity in RA-ILD has been associated with reduced survival and with acute exacerbation of ILD in previous observational studies. Elevated CDAI scores have been linked to increased mortality [13], moderate-to-high DAS28-ESR scores have been associated with poorer outcomes even among patients with preserved lung function [14], and CDAI > 10 during tocilizumab therapy has been reported as a risk factor for ILD exacerbation [38]. Our findings are compatible with these reports, but we did not assess pulmonary outcomes, survival, or treatment effects, and our results should not be read as evidence that controlling disease activity improves these outcomes.

The practical implication of our findings is modest but concrete. Baseline disease activity, which is measured at every routine visit, identifies patients with RA-ILD who are considerably less likely to be in remission or low disease activity 6 months later. Whether this information should alter the frequency of follow-up or the timing of reassessment was not tested in the present study. Our data do not indicate which treatment should be selected for these patients, nor do they show that any particular strategy improves articular or pulmonary outcomes; answering those questions requires studies designed to compare treatment strategies.

### Limitations

This study has several limitations. It was a retrospective analysis of an observational registry, and unmeasured confounding cannot be excluded. Baseline DAS28-ESR was missing in 100 of the 278 patients, so the models were fitted on 178 complete cases. These 178 patients and the 100 in whom the baseline DAS28-ESR was missing did not differ appreciably in age, sex, disease duration, smoking history, RF or ACPA positivity, MMP-3, KL-6, baseline CDAI, or in the 6-month outcome itself (Table S2), but complete-case analysis may still introduce selection bias through characteristics that were not measured. A further 205 patients with RA-ILD were excluded for want of a 6-month outcome, and the registry does not record whether these patients died or were lost to follow-up. The model was validated internally by bootstrap resampling only; external validation in an independent cohort has not been performed. The cut-off values were derived from these data, and the age threshold in particular was unstable across analysis sets. The association between age and the outcome was not reproduced when an ESR-free index was used, which limits the interpretability of age as a clinical risk factor. ILD was ascertained locally at each centre without standardized radiological criteria or central adjudication. At two centres (28 patients), the imaging modality used for diagnosis was known for 27: ILD had been diagnosed by HRCT in 24 and by chest radiography alone in 3; at the remaining centres the modality could not be determined retrospectively. At some centres, the absence of ILD is determined from chest radiography alone in routine practice, so patients with radiographically occult ILD may not have been identified. This primarily affects the definition and generalizability of the RA-ILD study population and may also have introduced selection or misclassification bias. HRCT pattern, extent of fibrosis, and pulmonary function were not available, so ILD severity could not be taken into account, and whether patients with an overlapping connective tissue disease or those receiving home oxygen therapy were included could not be established, as neither was recorded in the registry. Baseline did not correspond to the initiation of treatment, and treatment changes during the 6-month interval were not captured. Patients were recruited at six centres over more than a decade, during which treatment strategies changed substantially, and the cohort was restricted to Japanese patients, which may limit generalizability. Finally, pulmonary outcomes and treatment effects were not evaluated.

## 5. Conclusions

In patients with RA-ILD, baseline disease activity was strongly associated with moderate-to-high disease activity at 6 months. The model including age and baseline DAS28-ESR showed little estimated optimism on bootstrap internal validation (optimism-corrected area under the curve 0.772), although the optimism-corrected calibration slope of 0.984 was estimated with a wide confidence interval (0.693–1.396). The incremental contribution of age was small and was not reproduced when the outcome was defined by the CDAI. The age finding should therefore be regarded as secondary and dependent on the disease activity measure used. External and prospective validation is required before these findings are applied clinically.

## Figures and Tables

**Table 1:** Baseline clinical characteristics and initial treatments in patients with RA-associated interstitial lung disease (RA-ILD) from the ANSWER cohort (*N* = 278).

Characteristic	Patients with RA-ILD (*N* = 278)
Age, years	71 (64–76)
Female sex (%)	68.0
Disease duration of RA, years	14.4 (8.3–23.5)
Smoking history (%)	43.1
Steinbrocker stage (I/II/III/IV)	29.8/26.0/19.4/24.8
Steinbrocker class (I/II/III/IV)	27.2/49.2/19.7/3.9
HAQ (0–3)	0.75 (0.25–1.375)
Positive for RF (%)	84.6
RF, IU/mL	91 (29.7–226)
Positive for ACPA (%)	85.1
ACPA, U/mL	130 (16.3–444.3)
MMP-3, ng/mL	110.7 (60.7–211.6)
KL-6, U/mL	411 (241–687)
DAS28-CRP	2.92 (1.88–4.34)
DAS28-ESR	3.94 (2.80–5.15)
SDAI	11.9 (4.4–20.6)
CDAI	10.4 (4.2–18.9)
MTX (%)	23.4
MTX dose, mg/week	8.0 (6.0–10.0)
PSL (%)	35.3
PSL dose, mg/day	5.0 (3.0–5.6)
SASP (%)	25.2
BUC (%)	6.8
TAC (%)	16.5
IGU (%)	11.9
TNFi (%)	6.5
IL-6Ri (%)	10.8
CTLA4-Ig (%)	6.1
JAKi (%)	6.5

Continuous variables are presented as median (interquartile range), and categorical variables as percentages. RA, rheumatoid arthritis; ILD, interstitial lung disease; HAQ, Health Assessment Questionnaire; RF, rheumatoid factor; ACPA, anti-cyclic citrullinated peptide antibody; MMP-3, matrix metalloproteinase-3; KL-6, Krebs von den Lungen-6; CRP, C-reactive protein; ESR, erythrocyte sedimentation rate; DAS28, Disease Activity Score in 28 joints; SDAI, Simplified Disease Activity Index; CDAI, Clinical Disease Activity Index; MTX, methotrexate; PSL, prednisolone; SASP, salazosulfapyridine; BUC, bucillamine; TAC, tacrolimus; IGU, iguratimod; TNFi, tumor necrosis factor inhibitors; IL-6Ri, interleukin-6 receptor inhibitors; CTLA4-Ig, cytotoxic T-lymphocyte-associated protein 4 immunoglobulin; JAKi, Janus kinase inhibitors.

**Table 2:** Comparison of baseline characteristics between patients who achieved remission or low disease activity (LDA) and those with moderate-to-high disease activity (MDA/HDA) at 6 months.

Characteristics and Treatments	Remission/LDA Group (*n* = 148)	MDA/HDA Group (*n* = 130)	*p*-Value
Age, years	69 (62.5–75)	72 (65–78)	0.0182 *
Female, *n* (%)	91 (61.5)	98 (75.4)	0.0146 *
Smoking history, *n* (%) ^a^	61 (46.6)	48 (39.3)	0.2558
Disease duration of RA, years ^b^	12.6 (8.0–21.4)	15.5 (8.5–28.4)	0.0820
HAQ (0–3) ^c^	0.5 (0–1)	1.06 (0.63–1.97)	<0.001 ***
RF positive, *n* (%) ^d^	109 (79.0)	110 (90.9)	0.0095 **
RF, IU/mL ^d^	67.4 (20–178.4)	108.8 (45.5–311.9)	0.0057 **
ACPA positive, *n* (%) ^e^	101 (83.5)	87 (87.0)	0.5703
ACPA, U/mL ^e^	125 (14.6–411.5)	136.5 (22.7–485.8)	0.5398
MMP-3, ng/mL ^f^	91.3 (51.5–174.0)	155.7 (71.9–258.5)	0.0019 **
KL-6, U/mL ^g^	384 (233–776.5)	420 (267–687)	0.4521
DAS28-CRP ^h^	2.05 (1.54–3.4)	3.86 (2.69–4.84)	<0.001 ***
DAS28-ESR ^i^	3.06 (2.03–4.22)	4.79 (3.80–5.55)	<0.001 ***
SDAI ^j^	6.8 (2.5–14.6)	17.6 (10.7–26.2)	<0.001 ***
CDAI ^k^	6.0 (2.2–14.0)	14.6 (9.1–22.9)	<0.001 ***
MTX use, *n* (%)	39 (26.4)	26 (20.0)	0.2561
MTX dose, mg/week	8.0 (6–10)	7.0 (4–10)	0.5126
PSL use, *n* (%)	45 (30.4)	53 (40.8)	0.0790
PSL dose, mg/day	5.0 (2.8–10)	5.0 (3.0–5.0)	0.4536
TNFi, *n* (%)	10 (6.8)	8 (6.2)	1.0000
IL-6Ri, *n* (%)	20 (13.5)	10 (7.7)	0.1265
CTLA4-Ig, *n* (%)	9 (6.1)	8 (6.2)	1.0000
JAKi, *n* (%)	13 (8.8)	5 (3.9)	0.1414

Continuous variables are expressed as median (interquartile range). *p*-values were calculated using Fisher’s exact test or the Mann–Whitney *U* test, as appropriate. * *p* < 0.05, ** *p* < 0.01, *** *p* < 0.001. Numbers of patients with available data: ^a^ 253; ^b^ 256; ^c^ 153; ^d^ 259; ^e^ 221; ^f^ 184; ^g^ 111; ^h^ 185; ^i^ 178; ^j^ 181; ^k^ 197. All other variables were available for all 278 patients. RA, rheumatoid arthritis; HAQ, Health Assessment Questionnaire; RF, rheumatoid factor; ACPA, anti-cyclic citrullinated peptide antibody; MMP-3, matrix metalloproteinase-3; KL-6, Krebs von den Lungen-6; CRP, C-reactive protein; ESR, erythrocyte sedimentation rate; DAS28, Disease Activity Score in 28 joints; SDAI, Simplified Disease Activity Index; CDAI, Clinical Disease Activity Index; MTX, methotrexate; PSL, prednisolone; TNFi, tumor necrosis factor inhibitors; IL-6Ri, interleukin-6 receptor inhibitors; CTLA4-Ig, cytotoxic T-lymphocyte-associated protein 4 immunoglobulin; JAKi, Janus kinase inhibitors.

**Table 3:** Univariable and multivariable logistic regression analyses of baseline factors associated with moderate-to-high disease activity at 6 months in patients with RA-ILD.

	Univariable Analysis				Multivariable Model	
Baseline Variable	OR	95% CI	*p*-Value	*n*	OR (95% CI)	*p*-Value
Age, per year	1.040	1.013–1.067	0.0029 **	278	1.046 (1.010–1.084)	0.0125 *
Baseline DAS28-ESR, per point	1.989	1.561–2.534	<0.0001 ***	178	2.006 (1.568–2.567)	<0.0001 ***
Female (ref: male)	1.918	1.142–3.222	0.0138 *	278	—	—
Disease duration, per year	1.026	1.004–1.049	0.0222 *	256	—	—
Smoking history (ref: never)	0.744	0.452–1.227	0.2469	253	—	—
HAQ, per point	2.537	1.628–3.954	<0.0001 ***	153	—	—
RF, per 10 IU/mL	1.015	1.005–1.025	0.0047 **	259	—	—
MMP-3, per 10 ng/mL	1.015	0.997–1.034	0.1012	184	—	—
CDAI, per point	1.087	1.050–1.125	<0.0001 ***	197	—	—

Odds ratios are for moderate-to-high disease activity at 6 months. The number of patients contributing to each model is given; all analyses were complete-case. The multivariable model included age and baseline DAS28-ESR as continuous variables. * *p* < 0.05, ** *p* < 0.01, *** *p* < 0.001. Ref, reference; CI, confidence interval; OR, odds ratio; HAQ, Health Assessment Questionnaire; RF, rheumatoid factor; MMP-3, matrix metalloproteinase-3; DAS28-ESR, Disease Activity Score in 28 joints using erythrocyte sedimentation rate; CDAI, Clinical Disease Activity Index.

**Table 4:** Discrimination of the three models and internal bootstrap validation (*n* = 178; 88 events).

Model	AUC	95% CI	Note
Age alone	0.598	0.514–0.679	—
Baseline DAS28-ESR alone	0.768	0.699–0.835	Reference model
Age + baseline DAS28-ESR (continuous)	0.776	0.700–0.846	LR test *p* = 0.0092
Internal validation (1000 bootstrap resamples)			
Apparent AUC	0.7761	—	—
Optimism	0.0046	—	—
Optimism-corrected AUC	0.7715	—	—
Optimism-corrected calibration slope	0.984	0.693–1.396	—

AUC, area under the receiver operating characteristic curve; CI, confidence interval; DAS28-ESR, Disease Activity Score in 28 joints using erythrocyte sedimentation rate. Confidence intervals were obtained from 2000 bootstrap resamples. The likelihood-ratio test compares the model containing baseline DAS28-ESR alone with the model containing both age and baseline DAS28-ESR. Optimism was estimated from 1000 bootstrap resamples; the apparent area, the estimated optimism, and the optimism-corrected area are given to four decimal places so that their relationship is apparent, whereas the model areas above are given to three. All models were fitted on the 178 patients with a complete baseline DAS28-ESR; the area under the curve for age reported in Figure S1, which relates to the derivation of the age cut-off, was obtained in all 278 patients and therefore differs slightly.

## Data Availability

The data presented in this study are available on request from the corresponding author. The data are not publicly available due to the need to protect patient privacy.

## Supplementary Materials

The following supporting information can be downloaded at: https://www.mdpi.com/article/10.3390/jcm15218267/s1, Table S1: Comparison of baseline characteristics between included and excluded patients; Table S2: Comparison of baseline characteristics between the 178 patients with a complete baseline DAS28-ESR and the 100 in whom it was missing; Figure S1: ROC curve analyses for baseline DAS28-ESR and age.

## Abbreviations

The following abbreviations are used in this manuscript:

RA	rheumatoid arthritis
ILD	interstitial lung disease
RA-ILD	rheumatoid arthritis-associated interstitial lung disease
ANSWER	Kansai Consortium for Well-Being of Rheumatic Disease Patients
DAS28-ESR	Disease Activity Score in 28 joints using erythrocyte sedimentation rate
DAS28-CRP	Disease Activity Score in 28 joints using C-reactive protein
SDAI	Simplified Disease Activity Index
CDAI	Clinical Disease Activity Index
LDA	low disease activity
MDA/HDA	moderate/high disease activity
HAQ	Health Assessment Questionnaire
RF	rheumatoid factor
ACPA	anti-cyclic citrullinated peptide antibody
MMP-3	matrix metalloproteinase-3
KL-6	Krebs von den Lungen-6
MTX	methotrexate
PSL	prednisolone
csDMARD	conventional synthetic disease-modifying antirheumatic drug
bDMARD	biological disease-modifying antirheumatic drug
TNF	tumor necrosis factor
IL-6	interleukin-6
CTLA4-Ig	cytotoxic T-lymphocyte-associated protein 4 immunoglobulin
JAKi	Janus kinase inhibitor
HRCT	high-resolution computed tomography
ROC	receiver operating characteristic
OR	odds ratio
CI	confidence interval
