# Supplementary material for: Baseline Disease Activity and Moderate-to-High Disease Activity at 6 Months in Rheumatoid Arthritis-Associated Interstitial Lung Disease: A Multicentre Real-World Study from the ANSWER Cohort
Source: J Clin Med. 2026 Oct 23;15(21):8267. doi: 10.3390/jcm15218267 (PMC13764567; doi:10.3390/jcm15218267)
Supplement: Supplementary file 1 [file jcm-15-08267-s001.zip › jcm-4541658-supplementary.pdf]

Journal of Clinical Medicine

MDPI

*Supplementary Materials*

# Baseline Disease Activity and Moderate-to-High Disease Activity at 6 Months in Rheumatoid Arthritis-Associated Interstitial Lung Disease: A Multicentre Real-World Study from the ANSWER Cohort

**Ayaka Yoshikawa [1], Takuya Kotani [1,*], Wataru Yamamoto [2], Kenichiro Hata [1], Koji Nagai [1], Hideyuki Shiba [1], Yuri Hiramatsu [1], Yumiko Wada [1], Koichi Murata [3], Hideo Onizawa [3], Kohei Tsujimoto [4], Iku Shirasugi [5], Masanori Tsubosaka [6], Motomu Hashimoto [7], Masao Katsushima [7], Yonsu Son [8], Naofumi Yoshida [8], Yuji Nozaki [9], Chisato Ashida [9] and Tohru Takeuchi [1]**

**Table S1.** Comparison of baseline characteristics between the 278 patients included in the analysis and the 205 patients with RA-ILD excluded because a 6-month DAS28-ESR value was unavailable.

| Characteristic | Included (*n* = 278) | Excluded (*n* = 205) | *p*-Value |
|---|---|---|---|
| Age, years | 71 (64–76) | 71 (65–76) | 0.668 |
| Female, *n* (%) | 189 (68.0) | 144 (70.2) | 0.620 |
| Disease duration of RA, years | 14.4 (8.4–23.5) | 12.9 (6.2–26.1) | 0.418 |

Continuous variables are expressed as median (interquartile range). *p*-values were calculated using the Mann–Whitney *U* test or the Fisher exact test, as appropriate. Disease duration was available in 256 included and 183 excluded patients. RA, rheumatoid arthritis; ILD, interstitial lung disease; DAS28-ESR, Disease Activity Score in 28 joints using erythrocyte sedimentation rate.

**Table S2.** Comparison of baseline characteristics between the 178 patients with a complete baseline DAS28-ESR, who constituted the regression sample, and the 100 patients of the analysis population in whom the baseline DAS28-ESR was missing.

| Characteristic | Baseline DAS28-ESR Available (*n* = 178) | Baseline DAS28-ESR Missing (*n* = 100) | *p*-Value |
|---|---|---|---|
| Age, years | 70 (63–76) | 72 (66–76) | 0.172 |
| Female, *n* (%) | 119 (66.9) | 70 (70.0) | 0.688 |
| Disease duration of RA, years [a] | 14.5 (7.8–25.5) | 12.5 (9.0–21.5) | 0.713 |
| Smoking history, *n* (%) [b] | 71 (41.8) | 38 (45.8) | 0.590 |
| RF positive, *n* (%) [c] | 146 (86.4) | 73 (81.1) | 0.282 |
| ACPA positive, *n* (%) [d] | 127 (87.6) | 61 (80.3) | 0.166 |
| MMP-3, ng/mL [e] | 111.7 (63.0–205.8) | 108.7 (58.2–239.6) | 0.875 |
| KL-6, U/mL [f] | 378 (224–622) | 445 (246–810) | 0.207 |
| CDAI [g] | 10.4 (4.1–18.9) | 10.6 (5.1–17.5) | 0.973 |
| MDA/HDA at 6 months, *n* (%) | 88 (49.4) | 42 (42.0) | 0.261 |

Continuous variables are expressed as median (interquartile range). *p*-values were calculated using the Mann–Whitney *U* test or the Fisher exact test, as appropriate. Numbers of patients with available data: [a] 157 and 99; [b] 170 and 83; [c] 169 and 90; [d] 145 and 76; [e] 129 and 55; [f] 66 and 45; [g] 173 and 24, respectively. RF, rheumatoid factor; ACPA, anti-cyclic citrullinated peptide antibody; MMP-3,

matrix metalloproteinase-3; KL-6, Krebs von den Lungen-6; CDAI, Clinical Disease Activity Index; MDA/HDA, moderate-to-high disease activity; DAS28-ESR, Disease Activity Score in 28 joints using erythrocyte sedimentation rate.

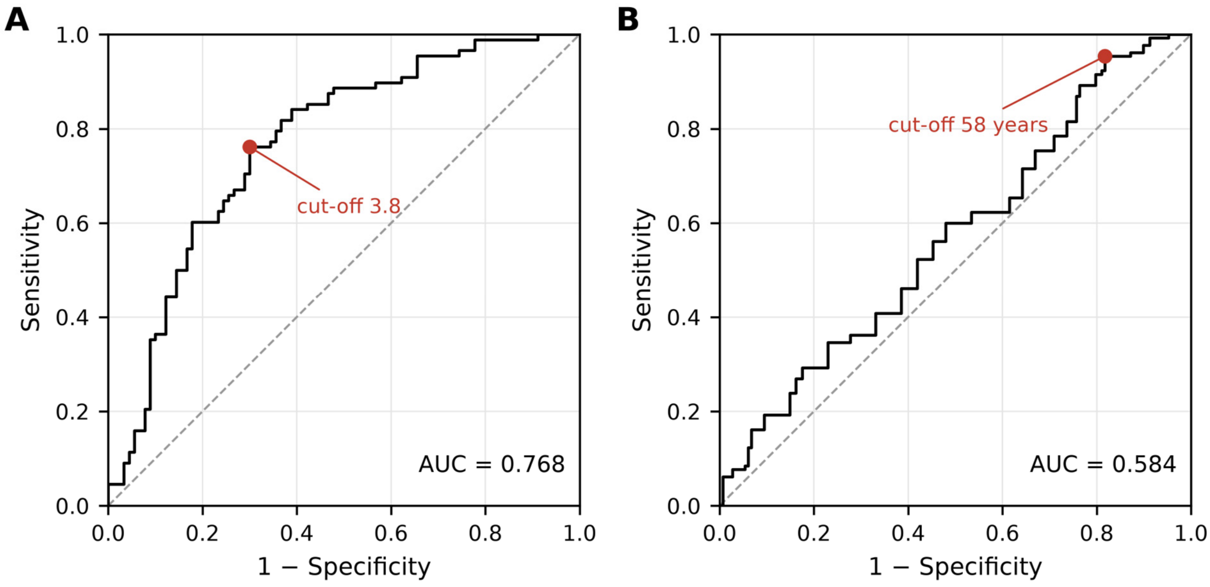


**Figure S1.** ROC curve analyses for predicting moderate-to-high disease activity (MDA/HDA) at 6 months in patients with RA-ILD. (**A**) Baseline DAS28-ESR demonstrated good discriminative ability (*n* = 178; AUC 0.768; cut-off 3.8; sensitivity 0.76; specificity 0.70). (**B**) Age showed limited predictive performance (*n* = 278; AUC 0.584; cut-off 58 years; sensitivity 0.95; specificity 0.18). DAS28-ESR, Disease Activity Score in 28 joints using erythrocyte sedimentation rate; MDA, moderate disease activity; HDA, high disease activity; RA-ILD, rheumatoid arthritis-associated interstitial lung disease; AUC, area under the curve; ROC, receiver operating characteristic.
